# Contraceptive availability leads to increase in use in conflict-affected Democratic Republic of the Congo: evidence from cross-sectional cluster surveys, facility assessments and service statistics

**DOI:** 10.1186/s13031-017-0104-2

**Published:** 2017-03-08

**Authors:** Sara E. Casey, Martin Tshipamba

**Affiliations:** 1 RAISE Initiative, Heilbrunn Department of Population and Family Health, Mailman School of Public Health, Columbia University, 60 Haven Ave, New York, NY 10032 USA; 2 SAF-PAC Project, CARE, 65, Av.de la corniche, Quartier les Volcans, Goma, Nord Kivu Democratic Republic of the Congo

**Keywords:** Contraception, War, Democratic Republic of the Congo, Humanitarian aid, Reproductive health

## Abstract

**Background:**

Humanitarian assistance standards mandate specific attention to address the sexual and reproductive health (SRH) needs of conflict-affected populations. Despite these internationally recognised standards, access to SRH services is still often compromised in conflict settings. CARE in collaboration with the RAISE Initiative strengthened the Ministry of Health (MOH) to provide contraceptive services in Maniema province, Democratic Republic of the Congo. This study evaluated the effectiveness of this support for MOH health facility provision of contraception.

**Methods:**

Cross-sectional surveys in 2008 (*n* = 607) and 2010 (*n* = 575) of women of reproductive age using a two-stage cluster sampling design were conducted in Kasongo health zone. Facility assessments were conducted to assess the capacity of supported government health facilities to provide contraceptive services in 2007 and 2010. Data on the numbers of clients who started a contraceptive method were also collected monthly from supported facilities for 2008–2014.

**Results:**

Current use of any modern contraceptive method doubled from 3.1 to 5.9% (adjusted OR 2.03 [95%CI 1.3–3.2]). Current use of long-acting and permanent methods (LAPM) increased from 0 to 1.7% (*p* < .001), an increase that was no longer significant after adjustment. All current users except a few condom users reported a health facility as the source of the method. The 2010 facility assessments found that most supported facilities had the capacity to provide short-acting and long-acting methods. Service statistics indicated that the percentage of clients who accepted a long-acting method at supported facilities increased from 8% in 2008 to 83% in 2014 (*p* < .001).

**Conclusions:**

This study demonstrated that contraceptive prevalence doubled between 2008 and 2010; service statistics indicate that utilization of long-acting methods continued to increase to a majority of new clients after 2010. Strengthening the health system to provide contraceptive services enabled individuals to exercise their right to prevent unintended pregnancies. These results suggest that demand for contraception, including long-acting methods, is present even in humanitarian settings, and that women will use them when they are available and of reasonable quality. It is critical that the humanitarian community ensure that such services are available to women affected by crises.

## Background

Complex humanitarian emergencies caused by armed conflict devastate already weak national health systems through the destruction of health facilities and flight of trained health workers [1]. Women living in conflict and post-conflict settings may face many sexual and reproductive health (SRH) concerns including high risk of mortality or morbidity due to pregnancy-related causes, unintended or unwanted pregnancy due to lack of information or access to contraceptive services, complications of unsafe abortions, gender-based violence and sexually transmitted infections including HIV [2, 3]. The ten countries with the highest maternal mortality ratios in the world are affected by, or emerging from, war; these countries are also characterized by low contraceptive prevalence [4, 5]. Minimum standards of humanitarian assistance now recognize this increased risk and require attention to the SRH needs of the population [6]. Despite this, the availability of contraceptive services and information is still limited in humanitarian settings [7, 8] although demand for birth spacing or limiting exists in conflict-affected populations [9]. Few humanitarian organizations have prioritized contraception, especially long-acting and permanent methods, while SRH agencies rarely work in humanitarian settings [2, 8]. When such services are available in humanitarian settings, they often are limited to short-acting methods [10–12].

Implementing comprehensive contraceptive services reduces the number of maternal deaths, particularly in countries with low contraceptive prevalence [13]. Maternal mortality and contraceptive prevalence have a strong negative correlation indicating that contraceptive services are a key intervention to prevent maternal mortality [13, 14]. According to the Countdown to 2015 for Maternal, Newborn and Child Survival, a reduction in maternal mortality and morbidity requires, among other changes, increased coverage of comprehensive contraceptive services [15, 16]. To address personal preferences and respond to changing clinical needs over the life course, a broad range of methods is an essential component of good contraceptive programming [17–20]. Previous research documents that when contraceptive method choice expands, prevalence usually increases [21, 22] perhaps because it provides more options or greater ability to meet women’s and couples’ individual needs; this appears to be true even in humanitarian settings [23].

### Context and program description

Nearly two decades of conflict and instability in eastern Democratic Republic of the Congo (DRC) have resulted in a compromised health system. Between 1998 and 2004, the conflict led to an estimated 3.9 million excess deaths [24]; the crude mortality rate was more than 70% higher than pre-war levels [25]. DRC has the sixth highest maternal mortality ratio in the world at 730 maternal deaths per 100,000 live births and a lifetime risk of maternal death of one in 23 [5]. The World Health Organization (WHO) determined that DRC made ‘insufficient progress’ towards achieving the fifth millennium development goal of improving maternal health [5]. For example, the DRC government contributes less than 1% of the cost of procuring contraceptives compared to 1–60% among other African countries [26, 27]. An index measuring the efforts of national contraceptive programs ranked DRC among the ten lowest performing countries in the world [28]. Modern contraceptive prevalence remains low at 7.8% in 2013, a small increase from 2007 (5.8%) [29, 30]. Since 2012, the DRC government has made a greater commitment to contraceptive services; however, this has yet to diffuse to the provincial level, especially the conflict-affected eastern provinces [26].

Kasongo, in southern Maniema province, was heavily affected by the conflict in the late 1990s and early 2000s and subsequent population displacement. The conflict exacerbated Kasongo’s already substantial isolation by halting river and railroad traffic; the poor roads fell further into disrepair. CARE International began supporting Ministry of Health (MOH) health services in Kasongo health zone in 2002, including limited contraceptive services: short-acting methods in all government health facilities and IUDs in two facilities beginning in 2004. According to current DRC MOH policy, health centres are mandated to provide all short- and long-acting reversible methods while referral hospitals are expected to also provide permanent methods. In late 2007, CARE partnered with the Reproductive Health Access, Information and Services in Emergencies (RAISE) Initiative [31] to improve SRH services in the referral hospital and all 21 government health centers in Kasongo health zone. The initial focus of the program was on improving emergency obstetric care; short and long-acting reversible contraceptive methods were reinforced or introduced in the 22 government health facilities in mid-2009. Tubal ligation was available at the hospital, primarily conducted during caesarean sections, although it was not as strongly reinforced as other methods; vasectomy was not available.

According to Bruce’s quality of care framework, essential elements of good quality contraceptive services include clinical competence of providers, counselling skills including the information given to clients, interpersonal skills, support for continuation of method use and integration with other health services [17]. All CARE support was coordinated with the MOH and included these essential components: competency-based clinical training for providers, training on contraceptive counselling, provision of essential equipment and supplies and monitoring and evaluation. Mechanisms to improve continuation of method use and follow-up of short-acting method users were put in place. In addition, support was provided to the MOH to improve supply chain management, and revise contraceptive services registers to collect relevant data. In 2011, CARE’s program shifted to focus on quality improvement and provided more in-depth support for contraceptive services in a smaller number of facilities (nine) in Kasongo, due to funding limitations [32].

All CARE support was coordinated through the MOH thus strengthening the health system, an important component of post-conflict recovery [1]. Health system strengthening is a long term process; for example, improving the MOH logistics system took time. The program focused first on establishing good quality contraceptive services, and less emphasis was placed on community education and outreach activities to avoid increasing demand for contraception before services were in place. Greater emphasis was appropriately placed on community education after 2010 when CARE established more systematic supervision and support of community educators, established expectations and regularly discussed challenges they faced and how to overcome them. Throughout the program, CARE worked closely with the MOH zonal team to strengthen their capacity to provide supportive supervision at the facilities in their health zone. For example, CARE and MOH supervisors worked with providers to identify weaknesses in service provision and jointly develop plans to address them. Together, they improved training on counseling with adaptations from the Population Council’s *Balanced counseling strategy* and the World Health Organization’s *Decision-making tool for family planning clients and providers* which use a series of job aids and an algorithm to promote unbiased contraceptive counseling [33, 34].

Few data are available to guide the effective provision of contraceptive services in conflict-affected settings, and the challenges to collecting data in these settings are well-recognised [35, 36]. In this paper, we discuss the results of cross-sectional population-based surveys, facility assessments conducted in 2007 and 2010 and monthly service statistics to evaluate the effectiveness of the health facility provision of contraception, by the MOH with CARE support, in Kasongo health zone.

## Methods

### Survey design & sample

Two cross-sectional population-based surveys were conducted in Kasongo health zone in February 2008 and October/November 2010. A two-stage cluster sampling design was used to ensure representation of the health zone population. Sampling was based on a 95% confidence interval and 50% contraceptive prevalence, the most conservative estimate which requires the largest sample size [37]. Anticipating an 80% response rate, a total of 625 households was selected in 2008 to achieve the required sample of 500 women; in 2010, expecting a response rate similar to the 97% achieved in 2008, 575 households were selected. Using MOH population estimates for villages in the catchment areas of each health facility, 25 clusters were selected using probability proportional to size. Within each cluster, 25 (2008) or 23 (2010) households were systematically selected using one of two methods. In larger villages, the study team randomly chose a direction from the center of the village, randomly selected the starting household from the first ten houses in that direction, and then systematically selected every tenth house until they reached the edge of the village. In villages with less than 250 households, the total number was divided by the desired number of households to determine the sampling interval; a random starting point was selected and then every n^th^ household based on this sampling interval. In 2008, after selection of the 25 clusters, two clusters were found to be inaccessible due to poor roads impassable because of rain; therefore, two additional clusters were selected. One woman of reproductive age (15–49 years) was selected from all eligible women in each household using a Kish table [38].

### Procedures

The survey questionnaire was adapted from the US Centers for Disease Control and Prevention’s *Reproductive Health Assessment Toolkit for Conflict Affected Women* [39]. The same questionnaire was used in 2008 and 2010 with minor adaptations to enhance clarity. The questionnaire covered multiple SRH topics; this paper focuses on the results related to contraception. The original CDC questionnaire was translated into French; all adaptations were made in the French version which was then translated into Congolese Swahili. The Swahili translation was subsequently reviewed and revised by the baseline survey team.

All interviewers (22 in 2008 and 16 in 2010) were female to ensure cultural appropriateness while increasing the likelihood of accurate data collection on SRH topics. During a ten-day training for supervisors and interviewers, they learned SRH terminology and survey techniques, and participated in practical exercises to assure mastery of material. The questionnaire was piloted in villages that were not included in the survey samples.

### Statistical analysis

Data were double-entered into CSPro Version 3.1 and subsequently exported to PASW (SPSS) Version 21 for cleaning and analysis. Data were weighted according to the number of eligible women of reproductive age in the household. Logistic regression was used to calculate odds ratios (ORs) and 95% confidence intervals to compare contraception outcomes in 2008 and 2010, adjusting for differences in the distribution of key population demographics which may influence contraception outcomes (education and religion in this study).

Modern contraceptive methods are defined as oral contraceptive pills, injectables, male and female condoms, implants, IUDs, tubal ligation, vasectomy. Long-acting methods are defined as implant and IUD; permanent methods as tubal ligation and vasectomy. Results are reported for all women, married and unmarried, unless otherwise specified. Regression models excluding unmarried women who were not sexually active produced the same results as those including all women.

### Facility assessments

To demonstrate that the supported health facilities had the capacity to provide the contraceptive services asked about in the survey, facility assessments were conducted in November/December 2007 and in July 2010 in 22 supported government health facilities. The trained assessment teams, which included MOH and CARE staff, used a standardized tool adapted from the Averting Maternal Death and Disability Program’s emergency obstetric care needs assessment tool [40]. The methods, including interviews with facility staff, a room by room inventory of equipment and supplies and clinical records review, evaluated physical infrastructure, human resources, infection prevention procedures and SRH service readiness [41]. Contraceptive service readiness was defined as the availability and functionality of essential equipment and supplies and presence of trained providers.

### Service statistics

Data on the numbers of clients who started a contraceptive method were collected monthly from the 22 government facilities from January 2008 through May 2011, and from nine government facilities June 2011 through December 2014.

### Ethical considerations

All interviewed participants were asked to give oral informed consent; names were not recorded to preserve anonymity. Parental consent for women aged 15–17 was waived since this study met the criteria for minimal risk. Ethical approvals for the study were obtained from the Institutional Review Board of the Mailman School of Public Health, Columbia University and the Congolese MOH.

## Results

### Surveys

The women in our 2008 and 2010 samples (607 and 564 respondents respectively) were similar with respect to age, marital or cohabitation status, ability to read, number of living children and having experienced an unwanted pregnancy (Table 1). Most respondents were under 35 years old, married, unable to read well or at all and had three or more children. Nearly all women reported being a local resident (rather than currently displaced). More women reported being Muslim in 2010 (73.1%) than in 2008 (66.4%), while fewer reported being Protestant in 2010 (3.7%) compared to 2008 (8.0%, *p* < .001). Although the percentage who reported being able to read easily remained the same, more women reported having at least some secondary education in 2010 (23.0%) than in 2008 (17.9%, *p* = .04).

**Table 1:** Socio-demographic characteristics of the populations sampled in 2008 and 2010

	2008 (*N* = 1006, 607)^a^	2010 (*N* = 1007, 564)^a^	*p*-value
Age (years)			*p* = .088
15–24	42.9% (264)	38.1% (215)	
25–34	31.4% (187)	33.6% (193)	
35–49	25.7% (155)	28.3% (156)	
Mean age (SD), years	28.0 (9.2)	28.8 (9.6)	*p* = .05
Marital status			*p* = .099
Married & living with husband	82.8% (517)	82.5% (486)	
Married & not living with husband	5.7% (38)	7.8% (34)	
Not married, living with partner	0.7% (3)	0.3% (2)	
Not married, not living with partner	10.8% (49)	9.3% (42)	
Number of living children			*p* = .416
0	18.3% (96)	16.6% (77)	
1–2	29.5% (199)	27.6% (168)	
3–4	27.7% (158)	29.0% (164)	
5+	24.5% (153)	28.3% (155)	
Religion			*p* < .001
Muslim	66.4% (394)	73.1% (404)	
Catholic	18.8% (115)	18.8% (107)	
Protestant	8.0% (55)	3.7% (23)	
Pentecostal/Evangelical	4.5% (29)	3.1% (24)	
Other	2.4% (14)	1.4% (6)	
Formal education			*p* = .037
None	35.6% (204)	34.1% (205)	
Did not complete primary school	35.6% (224)	32.4% (187)	
Completed primary school	10.9% (60)	10.5% (59)	
At least some secondary education	17.9% (119)	23.0% (113)	
Self-reported ability to read			*p* = .307
With difficulty or not at all	76.3% (453)	74.3% (434)	
Easily	23.7% (154)	25.7% (130)	
Number of lifetime pregnancies			*p* = .55
0	12.1% (57)	11.3% (54)	
1–2	21.5% (145)	22.3% (130)	
3–4	21.0% (129)	22.1% (128)	
5–9	34.7% (217)	31.9% (188)	
10+	10.7% (59)	12.3% (64)	
Reported an unwanted pregnancy			*p* = .419
No	73.9% (443)	75.6% (441)	
Yes	26.1% (163)	24.4% (123)	

Data are % of column weighted base (absolute counts), unless indicated. Bases are smaller for some variables due to missing data. Missing data are less than 0.5% for all variables

^a^N = weighted and unweighted base

Significant increases in four categories of knowledge and use of modern contraception between 2008 and 2010 are shown in Table 2. When asked to name any modern contraceptive method, 28.0% spontaneously mentioned at least one modern method in 2008 compared to 49.5% in 2010 (*p* < .001). In addition, 6.0% of women in 2008 spontaneously named a long-acting or permanent method (LAPM) while 10.3% of women did so in 2010 (*p* = .001). The percentage who reported having received instruction on how to use a modern method increased from 28.8% in 2008 to 44.5% in 2010 (*p* < .001), while those who reported having been instructed how to use an LAPM nearly doubled, from 7.9% in 2008 to 16.7% in 2010 (*p* < .001).

**Table 2:** Reported knowledge and use of modern contraceptive methods in 2008 and 2010

	2008 (95% CI) (*N* = 607)^a^	2010 (95% CI) (*N* = 564)^a^	*p*-value	Unadjusted OR (95% CI) (*N* = 1171)	Adjusted OR (95% CI)^b^ (*N* = 1171)	*p*-value, Adjusted OR
Spontaneous knowledge of modern contraceptive methods						
Any modern method	28.0% (25.1–30.7)	49.4% (46.3–52.6)	*p* < .001	2.5 (2.1–3.0)	2.7 (2.2–3.2)	*p* < 0.001
Any LAPM^c^	6.0% (4.4–7.6)	10.3% (8.5–12.2)	*p* = .001	1.8 (1.3–2.5)	1.87 (1.3–2.6)	*p* < 0.001
Reported prior instruction on how to use modern contraceptive method						
Any modern method	28.8% (26.2–31.7)	44.5% (41.4–47.5)	*p* < .001	1.98 (1.6–2.4)	1.98 (1.6–2.4)	*p* < 0.001
Any LAPM^c^	7.9% (6.2–9.5)	16.7% (14.5–19.1)	*p* < .001	2.3 (1.8–3.1)	2.2 (1.7–3.0)	*p* < 0.001
Reported ever use of modern contraceptive method						
Any modern method	11.5% (9.6–13.5)	18.8% (16.4–21.3)	*p* < .001	1.8 (1.3–2.8)	1.8 (1.4–2.4)	*p* < 0.001
Any LAPM^c^	0.3% (0.0–0.7)	2.0% (1.1–2.8)	*p* = .02			
Reported current use of modern contraceptive method						
Any modern method	3.1% (2.0–4.2)	5.9% (4.5–7.4)	*p* = .004	1.96 (1.3–3.0)	2.03 (1.3–3.2)	*p* = .002
Any LAPM^c^	0%	1.7% (1.0–2.6)	*p* < .001			

^a^N = unweighted base

^b^Adjusted for religion and education

^c^Long-acting and permanent methods (LAPM) are IUD, implant, tubal ligation and vasectomy

Ever use of any modern method increased from 11.5 to 18.8% (*p* < .001), while ever use of an LAPM increased from 0.3 to 2.0% (*p* = .02). Current use of a modern contraceptive method doubled from 3.1% in 2008 to 5.9% in 2010 (*p* = .004), while use of any LAPM increased from 0 in 2008 to 1.7% in 2010 (*p* < .001). The increase in current use of any modern method persisted when adjusted for religion and education (adjusted OR 2.03 [95% CI 1.3–3.2]). However, the increase in current use of a LAPM was no longer significant after adjusting for these socio-demographic variables. Increases in knowledge of LAPM (adjusted OR 1.87 [95% CI 1.3–2.6]) and having received instruction on how to use LAPM (adjusted OR 2.2 [95% CI 1.7–3.0]) persisted after adjustment. An adjusted model which excluded unmarried women who were not sexually active produced the same results as above.

Among women reporting current use of a modern contraceptive method in 2010, 81.1% reported a health facility as the source of the method while 19.0% of respondents, all of them condom users, reported a pharmacy or market as their source.

### Facility assessments

In 2007, all health facilities reported having provided pills and injectables in the three months prior to the assessment and 20 had the methods currently in stock; two reported providing IUDs and had them in stock, while no facility had implants or any staff trained to provide them (Table 3). In 2010, nearly all facilities had provided pills, injectables and implants in the three months prior to the assessment and had them in stock, while seven facilities had provided IUDs and nine had them in stock. All facilities had staff trained to insert and remove implants and 19 had staff trained to insert and remove IUDs in 2010.

**Table 3:** Number of facilities providing contraceptive services in 2007 and 2010

	2007 (*n* = 21)			2010 (*n* = 22)		
	Provided in previous 3 months (self-reported)	Method currently in stock	At least one staff trained to provide	Provided in previous 3 months (self-reported)	Method currently in stock	At least one staff trained to provide
Pills	21	20	21	22	21	22
Injectables	21	20	21	20	20	22
IUDs	2	2	2	7	9	19
Implants	0	0	0	21	21	22

### Service statistics

**Fig. 1:**
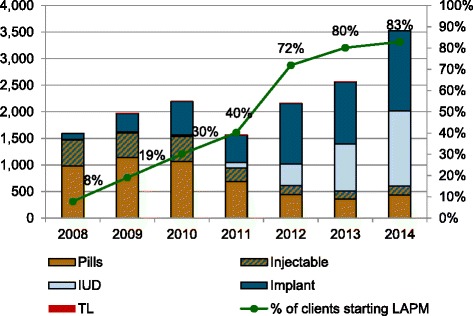
Number of clients who started contraceptive methods and percentage who started LAPM in health facilities supported by CARE in Kasongo, DRC (*n* = 22, 2008-May 2011 and *n* = 9, June 2011–2014)

**Fig. 2:**
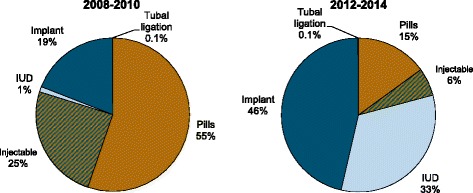
Contraceptive method mix from 2008–2010 (*n* = 22 facilities) and 2012–2014 (*n* = 9 facilities), Kasongo, DRC

From January 2008 to May 2011, the 22 facilities provided contraceptive methods to 9939 clients starting a method, including 2,202 who selected a LAPM (Fig. 1). The percentage of clients who accepted a LAPM increased from 7.9% in 2008 to 30.1% in 2010 (*p* < .001). From June 2011 to December 2014, 17,872 clients started a method at nine CARE-supported facilities, 13,708 of whom accepted a LAPM. The percentage of clients who accepted a LAPM increased dramatically to 82.9% in 2014 (*p* < .001). The method mix changed from dominance of short-acting methods in 2008–2010 to long-acting methods in 2012–2014; use of tubal ligation did not change (Fig. 2).

## Discussion

Our results demonstrate that increasing contraceptive use, even in remote conflict-affected settings, is feasible. Kasongo is an isolated, inaccessible region that was highly affected by the conflict of the late 1990s/early 2000s in eastern DRC. Knowledge of contraception was very low in 2008, with barely a quarter of respondents able to spontaneously name a modern contraceptive method, lower than the 70% found in rural areas nationally in the 2007 Demographic and Health Survey (DHS) [30]; only half were able to do so in 2010. Modern contraceptive prevalence in 2008 in Kasongo (3.1%) was similar to that found in rural DRC (3.3%) in the 2007 DHS. While contraceptive use remained low overall, prevalence nevertheless doubled from 2008 levels. The contraceptive prevalence of 5.9% found in this study was higher than the 3.6% found in rural areas of DRC in the 2010 MICS survey [42], as was the LAPM prevalence (1.7% versus 1.0%). We believe that the CARE program was the source of contraceptive method for most respondents who reported current use in 2010 as all except some condom users reported a public health facility as their source. This program supported contraceptive services at all of the public health facilities in the health zone; the 2010 facility assessments confirmed that contraceptive methods were available at all of these facilities; increasing numbers of clients accepted a method at these facilities during this time period. In addition, interviews with the MOH Health Zone Medical Officer indicated that no private facilities provided contraceptive methods, aside from condoms, in Kasongo at the time. Our results suggest that demand for contraception, including long-acting methods, is present even in humanitarian settings, and that women will use them when they are available and of reasonable quality.

While population-level survey data are important, people only use services that are available and of reasonable quality. Long-acting reversible contraceptives were new in Kasongo when they were introduced via this program, as demonstrated by the low knowledge of these methods and their near absence in facilities in 2008. The facility assessments found that long-acting methods were available in the supported facilities by 2010, and nearly all facilities had staff trained to provide both short- and long-acting methods. While all but one facility had implants in stock and reported providing implants in the three months prior to the 2010 assessments, just under half had IUDs in stock and had provided IUDs. While use of long-acting methods increased only slowly in the first two years of service provision, the service statistics show a dramatic change after 2010. Program adjustments were made to improve quality which likely contributed to the increased numbers of clients after 2010. The proportion of clients accepting a long-acting method increased from 8% of new acceptors in 2008 to 30% in 2010, and then to 84% in 2014. Program data demonstrate a steady and sharp increase in implant acceptors, from 115 new acceptors in 2008 to over 600 in 2010 and more than 1,500 in 2014. IUD acceptors, on the other hand, increased at a much slower rate early in the program, from 11 new acceptors in 2008 to 31 in 2010, and then to over 1,400 in 2014. This shift in method mix from dominance of short-acting methods to long-acting methods demonstrates that improving access to neglected methods, such as IUDs, is feasible. This study adds to the limited evidence that increasing method choice is associated with increased contraceptive use in isolated humanitarian settings.

These survey results led CARE to focus strongly on quality improvement in a smaller number of facilities in 2011: enhancing on-the-job training to improve counseling, address provider bias towards IUDs and ensure providers maintained clinical competency [32]. One barrier to the provision of LAPM, identified here and in similar programs, is limited provider competence [23, 43]. While all facilities had providers trained to provide long-acting methods, a potential reason for the slower increase in LAPM use from 2008–2010 could be related to provider confidence in their ability to provide these methods. Although efforts were made to ensure each provider had clinical practice during training, the low numbers of IUD acceptors, in particular, early in the program made it difficult for providers to maintain their skills or confidence in their skills, which may have discouraged them from offering these methods to clients. In response, supervisors carried anatomic pelvic models during supervision visits to permit providers to practice inserting IUDs under observation with a checklist which gave them greater confidence in their skills.

Other potential reasons for the initial limited increase in LAPM use from 2008–2010 may be bias against these methods, fear of side effects or misinformation regarding particular methods in the community [44]. To identify these potential barriers, CARE conducted a community analysis and developed education messages to address them. In addition to the program’s network of community educators, local community groups and satisfied users also conducted education on contraception. This more routine engagement allowed CARE to more quickly respond to rumors and misinformation in the community.

These survey results were used to address challenges related to provider bias and competence as well as barriers at community level. The increasing numbers of contraceptive clients starting a method in the supported health facilities, the availability of contraceptives in the supported facilities and lack of alternative sources of contraceptive services in Kasongo health zone, the increase in knowledge of contraceptive methods and increased ever and current use of contraception suggest successful implementation of this program [45, 46]. Although a third survey was not conducted, the service statistics suggest that this program has resulted in increasing utilization of contraception, and particularly of long-acting methods. The data indicate that while clients are interested in long-acting methods, barriers to IUD use among the community and providers took longer to address. Although these results may not be generalizable to all humanitarian settings, organizations implementing contraceptive services in such settings may find them useful. The positive results took time to achieve but are more likely to be sustained as all program components were implemented in collaboration with the MOH, including training, supervision and supplies management. Multi-year donor funding permitted this program to focus on quality improvement once the basic services were in place. Making good quality contraceptive services available was challenging and required sustained commitment, time and program adjustments, but was ultimately successful. It is important for other programs implementing contraceptive services in humanitarian settings to document how they achieved their success.

### Limitations

Data were collected in two cross-sectional surveys in 2008 and 2010 rather than using control communities, a choice made for ethical reasons. In their absence, it is difficult to identify changes in contraceptive prevalence that would have occurred without this program. Villages that were inaccessible were excluded from the sampling frame which may have led to an overestimation of results. It was the perception of the interviewers that respondents were largely forthcoming during the interviews; nevertheless, as in all surveys, respondents may have modified their answers according to social norms or to their perceptions of interviewer expectations or experienced recall bias. Although DRC presents challenges common to many humanitarian settings, the results may not be generalizable to other humanitarian settings.

## Conclusion

In conflict-affected countries, contraceptive availability is often limited to short-acting methods or none at all. This study demonstrated that contraceptive prevalence doubled between 2008 and 2010. Service statistics suggest that utilization of long-acting methods continued to increase to a majority of new clients after 2010, when provider skills and counseling improved and the methods became routinely available. Strengthening the health system to provide contraception enables individuals to exercise their right to prevent unintended pregnancies while improving the long-term sustainability of these services. Given true choice, when a range of methods was routinely available, women were able to choose the method that best served their needs, increasingly long-acting methods. This study demonstrates that even in remote and unstable settings like Kasongo, when good quality contraceptive services are in place, women will choose to use them. It is critical that the humanitarian community ensure that such services are available to women affected by crises.

## Abbreviations

DRC: Democratic Republic of the Congo
IUD: Intra-uterine device
LAPM: Long-acting or permanent method
MOH: Ministry of Health
OR: Odds ratio
RAISE Initiative: Reproductive Health Access, Information and Services in Emergencies
SRH: Sexual and reproductive health
WHO: World Health Organization
